# Incidental Unilateral Lateral Cortical Hypertrophy of the Proximal Femur Following Low‐Energy Trauma Without Prior Bisphosphonate Exposure: A Case Report

**DOI:** 10.1155/cro/6766372

**Published:** 2026-03-25

**Authors:** Shuichi Miyamoto, Yuichi Yoshii, Toshinori Tsukanishi, Kentaro Mataki, Toru Uchida, Tomohiko Inomoto, Tomoo Ishii

**Affiliations:** ^1^ Department of Orthopedic Surgery, Tokyo Medical University Ibaraki Medical Center, Ami-Machi, Ibaraki, Japan, tokyo-med.ac.jp

**Keywords:** atypical femoral fracture, bisphosphonate naïve, bone remodeling, cortical hypertrophy, proton pump inhibitors

## Abstract

Prolonged treatment with bisphosphonates has commonly been associated with atypical femur fractures. Localized periosteal thickening of the femoral lateral cortex is considered one of the minor features of these fractures and is caused by distribution of tensile stress and the inhibition of bone resorption. Other medications also potentially influence bone metabolism by inhibiting bone resorption. Here, we present a 60‐year‐old female with unilateral lateral cortical hypertrophy of the proximal femur with no history of bisphosphonate therapy. Long‐term administration of proton pump inhibitors may contribute to cortical hypertrophy in patients with low–bone‐turnover osteoporosis attributable to deficiencies in vitamins D and K. Two years after the patient′s initial visit to our hospital, radiographs demonstrated a reduction in periosteal thickening along the lateral cortex of the proximal femoral diaphysis. In a bisphosphonate‐naive patient with lateral cortical hypertrophy of the proximal femur, proton pump inhibitors′ use may represent a contributing factor that warrants consideration due to its potential association with disturbances in bone metabolism.

## 1. Introduction

Bone turnover is generally regarded as a process that maintains bone homeostasis through bone remodeling, involving the interplay between osteoclasts and osteoblasts [1, 2]. During bone remodeling, old or damaged bone is resorbed by osteoclasts and replaced with new bone formed by osteoblasts. Bisphosphonates (BPs) are widely used for treating osteoporotic fragility fractures and have been proven effective [3]. Their mechanism of action involves an inhibitory effect on bone resorption by acting on osteoclasts to suppress their activity or to increase the rate of apoptosis [4, 5]. Lateral cortical thickening on radiographs, suggestive of chronic stress reactions, has been observed in the subtrochanteric region of patients following long‐term BP use [6]. Over time, the accumulation of these reactions led to microscopic fractures, which eventually progressed to complete fractures [6, 7].

We present a case of unilateral lateral cortical hypertrophy of the proximal femur, identified incidentally after minor trauma, in the absence of BP treatment. Deficiencies of vitamins D and K, without evidence of secondary hyperparathyroidism, and prolonged use of proton pump inhibitors (PPIs) are suspected to influence bone metabolism and contribute to the occurrence of these types of radiographic findings, and we investigate whether it should be considered as a differential diagnosis.

## 2. Case Presentation

**Figure 1:**
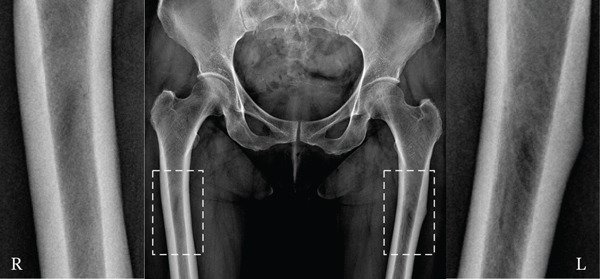
Anteroposterior radiographs of the bilateral hip joints and femurs at the initial consultation.

**Figure 2:**
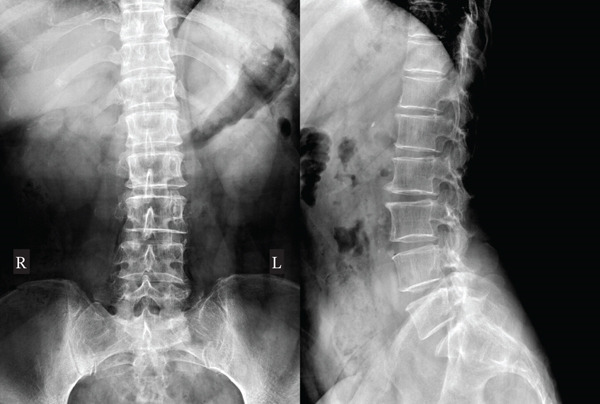
Anteroposterior and lateral radiographs of the lumbar spine.

A 60‐year‐old woman initially visited a neighborhood orthopedic clinic reporting right thigh pain following a stumble and fall. She was subsequently referred to our hospital for a comprehensive examination of an anomalous femoral finding detected on radiographs. She was able to walk independently without limping and her thigh pain decreased by the time she visited our hospital. An anteroposterior radiograph of the left proximal femoral diaphysis revealed a localized periosteal thickening of the lateral cortex (Figure 1). An anteroposterior radiograph of the lumbar spine demonstrated degenerative scoliosis with leftward convexity, the apex of which was at the L1–L2 level (Figure 2). On axial computed tomography (CT), the maximum lateral cortical thickness at the subtrochanteric level measured 8.79 mm on the right side and 11.34 mm on the left side (Figure 3a). CT was performed using a 320‐row multidetector CT scanner (Aquilion ONE 320 Slice; Canon Medical Systems, Tochigi, Japan) with a standardized imaging protocol (120 kVp, 264 mA, and 2.0‐mm slice thickness). Asymmetry of the lateral femoral cortical thickness was evaluated using axial CT images acquired along a plane connecting the inferior margins of the bilateral teardrops. Measurements were obtained at the subtrochanteric level, specifically at the site where periosteal thickening was most pronounced. To assess the reproducibility of the measurements, two board‐certified orthopedic surgeons independently measured the values on two separate occasions. Intra‐rater and interrater reliability were evaluated using the intraclass correlation coefficient (ICC), yielding values of 0.80 and 0.76, respectively. Magnetic resonance imaging (MRI) revealed no increased signal intensity around the subtrochanteric region of the left proximal femoral diaphysis, whereas increased intensity was observed in the subcutaneous adipose tissue (Figure 3b). The patient had been taking PPIs for approximately 5 years and had never taken BPs. Specifically, she had been taking esomeprazole (Nexium) 20 mg/day for the treatment of gastroesophageal reflux disease. Laboratory examination at the time of admission indicated a slightly elevated concentration of undercarboxylated osteocalcin (ucOC) and a deficiency (< 20 ng/mL) of serum 25‐hydroxyvitamin D (Table 1). Dual‐energy x‐ray absorptiometry (DXA) of the lumbar spine and total hip demonstrated bone mineral density (BMD) values of 0.743 and 0.535 g/cm3, with corresponding T‐scores of −2.3 and −2.8, respectively. Compared with a young adult reference population, the lumbar spine T‐score indicated osteopenia, and the total hip T‐score met the criteria for osteoporosis [8, 9]. The patient′s PPI medication was discontinued following the hospital visit. Her symptoms, such as thigh pain, were absent 2 weeks after the injury, and her activities of daily living were unhindered. At the final follow‐up 2 years after the injury, radiographs demonstrated a reduction in periosteal thickening of the lateral cortex in the proximal femoral diaphysis, and no fractures had occurred (Figure 4).

Figure 3(a) Bilateral coronal and axial computed tomographic images of the hips and femurs and (b) coronal magnetic resonance images of the left hip and femur at the initial consultation on a T1‐weighted image (T1WI) and a short T1 inversion recovery (STIR) image.

**Figure:**
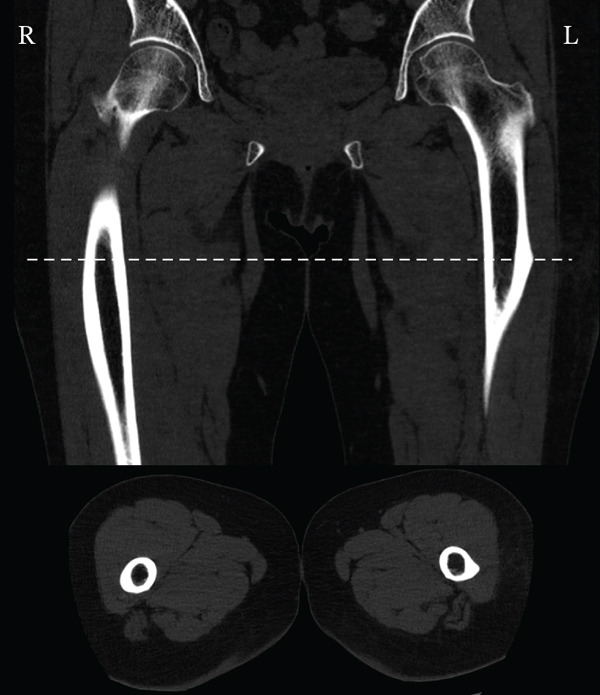
(a)

**Figure:**
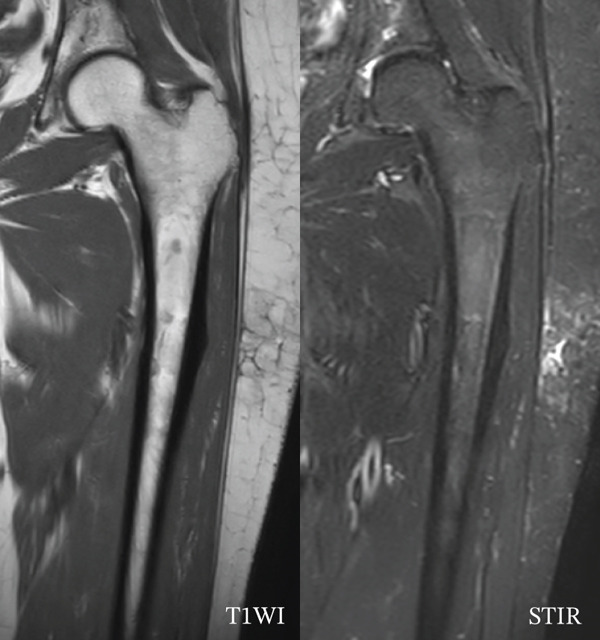
(b)

**Table 1:** Laboratory findings on hospital admission.

	Patient′s results
Blood cell counts	
White blood cells (/*μ*L)	7.8 × 10^3^
Neutrophils (%)	57.5
Lymphocytes (%)	32.1
Monocytes (%)	8.1
Eosinophils (%)	1.5
Basophils (%)	0.8
Red blood cells (/*μ*L)	4.48 × 10^6^
Hemoglobin (g/dL)	13.3
Hematocrit (%)	41.2
Platelet (/*μ*L)	312 × 10^3^

Biochemical tests	
Total protein (g/dL)	7.7
Albumin (g/dL)	4.3
Aspartate aminotransferase (IU/L)	20
Alanine transferase (IU/L)	29
Blood urea nitrogen (mg/dL)	11.2
Creatinine (mg/dL)	0.55
Na (mg/dL)	141
K (mg/dL)	4.1
Cl (mg/dL)	105
iPTH (pg/mL)	41
TRACP‐5b (mU/dL)	325
ucOC (ng/mL)	7.57
Total P1NP (ng/mL)	66.7
25(OH)D (ng/mL)	8.9

Immunochemistry	
CRP (mg/dL)	0.19

Abbreviations: 25(OH)D, 25‐hydroxyvitamin D; CRP, C‐reactive protein; iPTH, intact parathyroid hormone; P1NP, N‐terminal propeptide of Type I collagen; TRACP‐5b, tartrate‐resistant acid phosphatase 5b; ucOC, undercarboxylated osteocalcin.

Figure 4Anteroposterior radiographs of the left hip and femur (a) at the initial consultation and (b) 2 years later.

**Figure:**
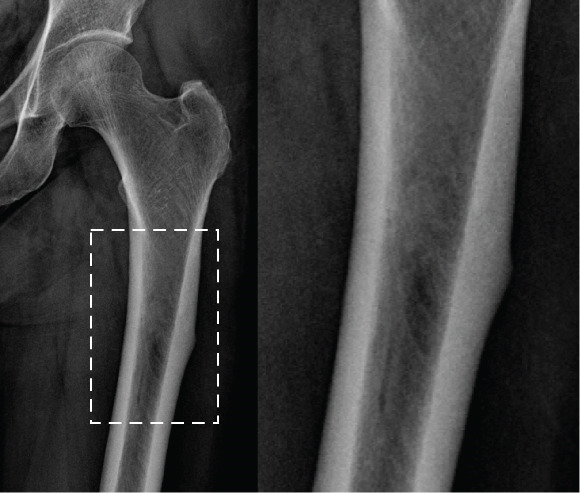
(a)

**Figure:**
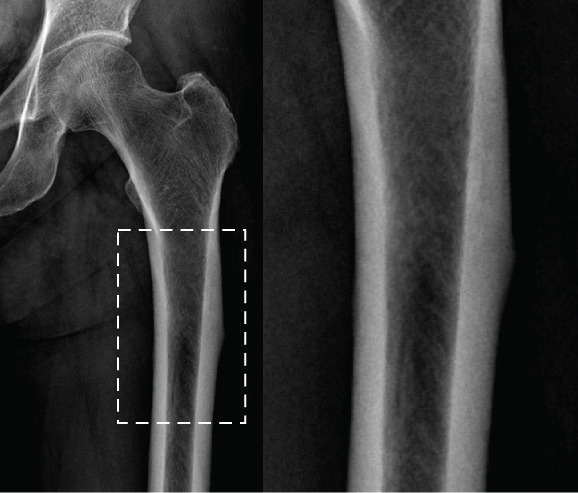
(b)

## 3. Discussion

Localized periosteal thickening of the femoral lateral cortex is one of the minor features of atypical femoral fractures (AFFs) [10], and therefore, the initial treatment received is crucial because of the potential risk of progression. BPs are commonly used to prevent the risk of osteoporotic fragility fractures based on their ability to inhibit osteoclast activity [3–5]. Conversely, prolonged BP therapy can lead to AFF [11, 12]. Bone turnover is a dynamic and continuous biological process essential for maintaining bone homeostasis. Osteoclasts and osteoblasts function collaboratively to regulate bone remodeling, which is essential for maintaining physiological bone homeostasis [1, 2]. This remodeling process consists of five distinct, sequential phases [13, 14]:

1. Activation—involves the differentiation of osteoclast precursors into mature osteoclasts, triggered by the repair of microdamage in the bone or the body′s metabolic demands.
2. Resorption—activated osteoclasts begin to dissolve and absorb old or damaged bone tissue, breaking down the bone matrix.
3. Reversal—after resorption, reversal cells prepare the bone surface for new bone formation; they achieve this by removing undigested collagen that has been demineralized during resorption.
4. Formation—osteoblasts are activated to synthesize new bone tissue; they deposit an unmineralized matrix, known as osteoid, which is later mineralized to form mature, mineralized bone.
5. Quiescence—following bone formation, the surface of the bone is covered by bone‐lining cells, entering a resting state until the next remodeling cycle is initiated.

The mechanisms underlying cortical thickening in the subtrochanteric region of the femur of long‐term users of BPs involve the suppression of natural bone remodeling and reduced bone metabolism due to the inhibition of osteoclastic bone resorption. The exponential accumulation of bone microdamage over time and the alterations to mineral and matrix properties can occur due to increased mineralization because of suppressed bone turnover. This phenomenon is observed as an increased thickness of the cortex on radiographs [15, 16]. Currently, the location of cortical thickening has been clinically associated with the distribution of tensile stress in the femur during weight bearing. Hence, femoral bowing, neck angle, and width have been documented as potential contributing factors [17, 18]. Because the lateral cortex of the femur—particularly from the subtrochanteric area to the diaphysis—is exposed to tensile stress, a higher rate of bone remodeling is necessary in this region compared with other regions. Conversely, the subtrochanteric region is composed mainly of cortical bone [19], which has a lower bone turnover rate than cancellous bone due to differences in blood supply [20]. As a result, this region of cortical bone is more prone to cumulative bone microdamage and strongly influenced by the inhibition of bone resorption. Bilateral periosteal thickening of the femoral lateral cortex is considered a minor feature of AFFs [10]. In this case, the unusual unilateral presentation of AFFs is likely attributable to an imbalance in mechanical stress distribution resulting from the patient′s degenerative scoliosis with a mild leftward curvature.

Despite the strong, causative link between BPs and AFFs, reports of other studies have indicated that glucocorticoids and PPIs, which influence bone metabolism, may be associated with an increased risk of AFFs [21, 22]. Interestingly, a retrospective cohort analysis of the Swedish National Patient Register revealed that 22% of patients with AFFs had never used BPs [23]. Although it has been established that PPIs inhibit gastric H^+^‐K^+^‐ATPase, some studies suggested that PPIs also inhibit the osteoclastic proton transport system by preventing a vacuolar‐type ATPase proton pump from acidifying the resorption lacuna [24, 25]. Furthermore, research in a murine model of lipopolysaccharide‐induced inflammatory calvarial osteolysis indicated that PPIs inhibit the bone resorptive activity of mature osteoclasts as a result of suppressing receptor activator of nuclear factor‐*κ*B ligand (RANKL)‐induced osteoclastogenesis from bone marrow monocytic/macrophage cells [26]. Synthesizing the results of biochemical analyses, including bone turnover markers and DXA, this case was characterized by concomitant deficiencies of vitamins D and K, notably without findings indicative of secondary hyperparathyroidism. These clinical observations are suggestive of a pathophysiological state consistent with low‐turnover osteoporosis, defined by synchronized suppression of both bone resorption and formation. Seminal studies have demonstrated that vitamin D acts as a key regulator of bone remodeling by promoting the expression of RANKL via the vitamin D receptor expressed on osteoblasts, subsequently inducing osteoclastogenesis and bone resorption [27]. Consequently, under conditions of vitamin D deficiency in the absence of secondary hyperparathyroidism, suppressed RANKL expression is likely to result in impaired osteoclast differentiation and function. In addition to the patient characteristics observed in this case, the long‐term use of PPIs may have further contributed to suppression of bone resorption, thereby exacerbating a low‐bone turnover state. As a result, marked stagnation of bone remodeling was considered one of the factors contributing to the underlying pathogenesis. Additionally, elevated ucOC indicates insufficient vitamin K–dependent *γ*‐carboxylation of osteocalcin and may have been associated with impaired bone quality, including altered mineral–matrix interactions and mineral maturation [28, 29]. A reduction in the mechanical strength of bone under these metabolic conditions could accelerate the cumulative accumulation of microdamage, particularly in regions that require continuous bone remodeling, such as the lateral cortex of the femur.

A primary limitation of the present study is the paucity of evidence regarding the anatomical distribution of cortical hypertrophy associated with PPIs use, as published reports describing involvement of specific anatomical regions are scarce [30]. Hence, whether PPIs exert a site‐specific effect on the skeleton cannot be determined definitively at present, and further studies with larger cohorts and detailed anatomical assessments are needed to clarify this issue.

## 4. Conclusion

In the current case report, we present a case of unilateral lateral cortical hypertrophy of the proximal femur in a patient not exposed to BP therapy. After approximately 5 years of PPI use, radiographs obtained 2 years after discontinuation of PPIs revealed a reduction in the periosteal thickening of the lateral cortex. When such findings are detected incidentally, a clinical evaluation of bone metabolic status is warranted, and PPI use should be investigated as a potential contributing factor.

## Funding

No funding was received for this manuscript.

## Consent

The patient described in this paper has provided informed consent for her case report and radiographs to be published.

## Conflicts of Interest

The authors declare no conflicts of interest.

## Supporting information


**Supporting Information** Additional supporting information can be found online in the Supporting Information section. The CARE checklist (2013 version) for case reports has been completed and provided to ensure transparency and adherence to the reporting guidelines for case reports.

## Data Availability

Data sharing is not applicable to this article as no new datasets were generated or analyzed for the current study.
