# Supplementary material for: Incidental Unilateral Lateral Cortical Hypertrophy of the Proximal Femur Following Low‐Energy Trauma Without Prior Bisphosphonate Exposure: A Case Report
Source: Case Rep Orthop. 2026 Mar 25;2026:6766372. doi: 10.1155/cro/6766372 (PMC13325405; doi:10.1155/cro/6766372)
Supplement: Supplementary file 1 — Supporting Information Additional supporting information can be found online in the Supporting Information section. The CARE checklist (2013 version) for case reports has been completed and provided to ensure transparency and adherence to the reporting guidelines for case reports. [file CRO-2026-6766372-s001.pdf]

CARE case report guidelines

CARE Checklist of information to include when writing a case report

(cc) BY-NC-ND

Topic	Item	Checklist item description	Reported on Line
Title	1	The diagnosis or intervention of primary focus followed by the words "case report"	Page 1, Line4-5
Key Words	2	2 to 5 key words that identify diagnoses or interventions in this case report, including "case report"	Page 2, Line65-66
Abstract (no references)	3a	Introduction: What is unique about this case and what does it add to the scientific literature?	Page 2, Line53-54
	3b	Main symptoms and/or important clinical findings	Page 2, Line54-56
	3c	The main diagnoses, therapeutic interventions, and outcomes	Page 2, Line56-60
	3d	Conclusion—What is the main "take-away" lesson(s) from this case?	Page 2, Line60-63
Introduction	4	One or two paragraphs summarizing why this case is unique (**may include references**)	Page 3, Line94-99
Patient Information	5a	De-identified patient specific information.	Page 4, Line110-113
	5b	Primary concerns and symptoms of the patient.	Page 4, Line113-116
	5c	Medical, family, and psycho-social history including relevant genetic information	Page 5, Line144-146
	5d	Relevant past interventions with outcomes	Page 5, Line144-146
Clinical Findings	6	Describe significant physical examination (PE) and important clinical findings.	Page 4, Line114-116
Timeline	7	Historical and current information from this episode of care organized as a timeline	Page 4, Line110-114
Diagnostic Assessment	8a	Diagnostic testing (such as PE, laboratory testing, imaging, surveys).	Page 4, Line118-132
	8b	Diagnostic challenges (such as access to testing, financial, or cultural)	Not applicable.
	8c	Diagnosis (including other diagnoses considered)	Page 8, Line242-245
	8d	Prognosis (such as staging in oncology) where applicable	Not applicable.
Therapeutic Intervention	9a	Types of therapeutic intervention (such as pharmacologic, surgical, preventive, self-care)	Page 5, Line153-154
	9b	Administration of therapeutic intervention (such as dosage, strength, duration)	Page 5, Line153-154
	9c	Changes in therapeutic intervention (with rationale)	Not applicable.
Follow-up and Outcomes	10a	Clinician and patient-assessed outcomes (if available)	Not applicable.
	10b	Important follow-up diagnostic and other test results	Page 5, Line154-157
	10c	Intervention adherence and tolerability (How was this assessed?)	Not applicable.
	10d	Adverse and unanticipated events	Not applicable.
Discussion	11a	A scientific discussion of the strengths AND limitations associated with this case report	Page 8-9, Line219-273
	11b	Discussion of the relevant medical literature **with references**.	Page 6-9, Line169-273
	11c	The scientific rationale for any conclusions (including assessment of possible causes)	Page 7-9, Line199-267
	11d	The primary "take-away" lessons of this case report (without references) in a one paragraph conclusion	Page 10, Line288-293
Patient Perspective	12	The patient should share their perspective in one to two paragraphs on the treatment(s) they received	Not applicable.
Informed Consent	13	Did the patient give informed consent? Please provide if requested	Yes ☑ No ☐
